# Unsuccessful Men

**Published:** 1854-10

**Authors:** 


Unsuccessful Men.—I confess that increasing years bring with them an increasing respect for men who do not succeed in life, as those words are commonly used. Heaven is said to be a place for those who have not succeeded upon earth; and it is surely true that celestial graces do not best thrive and bloom in the hot blaze of worldly prosperity. Ill success sometimes arises from a superabundance of qualities in themselves good—from a conscience too sensitive, a taste too fastidious, a self-forgetfulness too romantic, a modesty too retiring. I will not go so far as to say, with a living poet, that "the world knows nothing of its greatest men," but there are forms of greatness, or at least of excellence, which "die and make no sign," there are martyrs that miss the palm, but not the stake; heroes without the laurel, and conquerors without the triumph.

